# Graphene-Based Tunable Polarization Conversion Metasurface for Array Antenna Radar Cross-Section Reduction

**DOI:** 10.3390/s24155044

**Published:** 2024-08-04

**Authors:** Yang’an Zhang, Yuxi Li, Yao Li, Xueguang Yuan, Xin Yan, Xia Zhang

**Affiliations:** The State Key Laboratory of Information Photonics and Optical Communications, Beijing University of Posts and Telecommunications, Beijing 100876, China; zhang@bupt.edu.cn (Y.Z.); 22-lyx@bupt.edu.cn (Y.L.); liyao98@bupt.edu.cn (Y.L.); yuanxg@bupt.edu.cn (X.Y.); xzhang@bupt.edu.cn (X.Z.)

**Keywords:** tunable, polarization conversion metasurface (PCM), graphene, radar cross-section (RCS)

## Abstract

A graphene-based tunable polarization conversion metasurface (PCM) was designed and analyzed for the purpose of reducing the radar cross-section (RCS) of array antennas. The metasurface comprises periodic shuttle-shaped metal patches, square-patterned graphene, and inclined grating-patterned graphene. By adjusting the Fermi energy levels of the upper (*μ*_1_) and lower (*μ*_2_) graphene layers, different states were achieved. In State 1, with *μ*_1_ = 0 eV and *μ*_2_ = 0.5 eV, the polarization conversion ratio (PCR) exceeded 0.9 in the bandwidths of 1.65–2.19 THz and 2.29–2.45 THz. In State 2, with *μ*_1_ = *μ*_2_ = 0.5 eV, the PCR was greater than 0.9 in the 1.23–1.85 THz and 2.24–2.60 THz bands. In State 3, with *μ*_1_ = *μ*_2_ = 1 eV, the PCR exceeded 0.9 in the 2.56–2.75 THz and 3.73–4.05 THz bands. By integrating the PCM with the array antenna, tunable RCS reduction was obtained without affecting the basic radiation functionality of the antenna. In State 1, RCS reduction was greater than 10 dB in the 1.60–2.43 THz and 3.63–3.72 THz frequency ranges. In State 2, the RCS reduction exceeded 10 dB in the 2.07–2.53 THz, 2.78–2.98 THz, and 3.70–3.81 THz bands. In State 3, RCS reduction was greater than 10 dB in the 1.32–1.43 THz, 2.51–2.76 THz, and 3.76–4.13 THz frequency ranges. This polarization conversion metasurface shows significant potential for applications in switchable and tunable antenna RCS reduction.

## 1. Introduction

Under the conditions of informationized warfare, stealth technology stands as the most pivotal aspect of military capability advancement [1,2,3]. Reducing the radar cross-section (RCS) holds profound implications for stealth technology. Traditional approaches to RCS reduction predominantly rely on shaping techniques and radar-absorbing materials (RAMs). Despite achieving a certain level of success, these methods often suffer from limitations, such as bandwidth constraints, excessive weight, and bulky volumes. In particular, when applied to antenna systems, they can severely impact the radiation performance of antennas. In recent years, the metasurface has emerged as a promising candidate for reducing the RCS of antennas [4,5,6,7,8,9,10,11,12,13,14].

Due to artificially arranged periodic or quasi-periodic structures, metasurfaces exhibit electromagnetic properties that are unattainable by natural surfaces, such as perfect absorption [15,16,17], anomalous reflection [18,19,20,21], and polarization conversion [22,23,24,25]. The RCS of an antenna can be effectively reduced by means of energy scattering cancellation using polarization conversion characteristics. In recent years, various polarization conversion metasurfaces (PCMs) have been proposed for RCS reduction. Dumbbell-shaped PCMs were employed in [26] to reduce the RCS of slot-coupled patch antennas. In [27], a checkerboard fishbone PCM placed above a slot array antenna achieved a 5 dB RCS reduction within the 6.0 to 18.0 GHz range. 

It is noteworthy that the aforementioned PCM exhibits stealth capability only within specific frequency bands, lacking tunability to meet the growing demand for multifunctional applications. To overcome this limitation, the introduction of a tunable material with adjustable performance into the device is required. Graphene, due to its dynamic tunability [28], rapid carrier mobility, and high optical transparency [29], possesses inherent properties enabling the dynamic control of the electromagnetic response of metasurfaces. Thus, it can adjust scattering characteristics in real time according to evolving operational requirements or environmental conditions, thereby offering the potential to realize tunable RCS reduction for antennas. However, up to date, graphene-based tunable PCMs for array antenna RCS reduction have been rarely reported.

In this study, a tunable PCM based on graphene material was designed and analyzed for RCS reduction in array antennas. The metasurface was composed of periodic shuttle-shaped metal patches, square-patterned graphene, and inclined grating-patterned graphene mounted above the array antenna. By altering the Fermi energy levels of the upper (*μ*_1_) and lower (*μ*_2_) graphene layers, the metasurface could achieve both in-band and out-of-band tunable antenna RCS reduction in the bands of 1.0–5.0 THz while maintaining basic radiation functions. This polarization conversion metasurface demonstrates significant potential for switchable and tunable antenna RCS reduction and related applications.

## 2. Design of Polarization Conversion Unit

**Figure 1:**
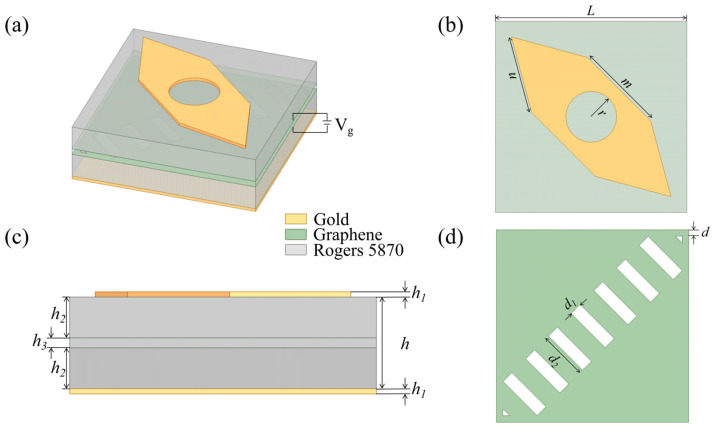
Specific structure of the polarization conversion unit. (**a**) Oriental view. (**b**) Top view. (**c**) Side view. (**d**) Inclined grating structure of the graphene layer.

The specific structure of the designed polarization conversion unit is depicted in Figure 1a as follows: the top layer of the metal, an intermediate dielectric layer incorporating graphene layers, and the bottom layer consisting of a metal reflector. The top and bottom layers are both fabricated from gold, possessing a conductivity of *σ* = 4.1 × 10^7^ S/m. The dielectric layer, situated between these metal layers, is constructed from Rogers 5870, characterized by a relative permittivity (*ε*) of 2.33 and a dielectric loss tangent of 1.2 × 10^−3^. As shown in Figure 1b, the top metal structure exhibits a shuttle-shaped configuration, with outer-edge dimensions denoted by *m* and *n* and an inner circular radius denoted by *r*. To better control polarization waves, this structure features a dual-layer graphene configuration. As illustrated in Figure 1c,d, the upper layer is designed with square patterns of side length *L*, while the lower layer is designed with inclined grating patterns. The optimized parameters are listed in Table 1.

### 2.1. Simulation Setup

The modeling and simulations of all structures in this study were conducted using Ansys HFSS. The simulation setup of the polarization conversion unit involved setting up master and slave boundaries in the x and y directions, while Floquet port excitation was employed in the z direction to simulate an infinite planar periodic array. A two-dimensional conductive surface was utilized to represent the graphene material, with the impedance boundary characterized by resistance and reactance.

### 2.2. Graphene Material

The surface conductivity of graphene is calculated using the Kubo formula [30,31], accounting for both intra-band and inter-band transitions. The specific formula is as follows:

(1) $$\sigma_{s}=\sigma_{intra}+\sigma_{inter}$$

(2) $$\sigma_{intra}=-i\frac{e^{2}K_{B}T}{\pi \hbar^{2}(\omega -i\tau^{-1})}(\frac{\mu }{K_{B}T}+2\ln(e^{-\mu /(k_{B}T)}+1))$$

(3) $$\sigma_{\mathrm{inter}}=\frac{-ie^{2}}{4\pi \hbar }\ln(\frac{2\left|\mu \right|-(\omega -i\tau^{-1})\hbar }{2\left|\mu \right|+(\omega -i\tau^{-1})\hbar })$$

In (2), *e* represents the elementary charge, *K_B_* denotes the Boltzmann constant, *ħ* stands for the reduced Planck constant (*ħ* = *h*/2π), *ω* represents angular frequency, and *μ* signifies the Fermi energy level of graphene. In simulations, *T* denotes the temperature, set to 300K, and *τ* represents the relaxation time, set to 1 ps.

The surface conductivity of graphene can be modulated by altering the Fermi energy level. By applying a transverse electric field through biased gate structures, *μ* can be adjusted within the range of −1 eV to 1 eV. Thus, the conductivity of graphene material can be controlled via a direct current bias voltage. The specific formula linking the two is as follows, when *E*_f_ >> *K_B_T* [28,29].

(4) $$\mu =E_{f}\approx \hbar v_{f}\sqrt{\frac{\pi \varepsilon_{r}\varepsilon_{0}V_{g}}{et_{s}}}$$

In (4), *v_f_* represents the Fermi velocity, set at 1.1 × 10^6^ m/s, *ε*_0_ denotes the vacuum permittivity, ε*_r_* signifies the relative permittivity, *V_g_* stands for the bias voltage, and *t_s_* represents the dielectric layer thickness. In summary, the approach of utilizing bias voltage to alter the graphene Fermi energy level, thereby dynamically controlling the electromagnetic response of the metasurface, is feasible.

### 2.3. Polarization Conversion Theory

Stacking multiple layers can extend the bandwidth of the polarization conversion unit and achieve tunability through the combined effects of each layer. The top and bottom metal layers serve distinct functions: the top layer acts as an electric field decomposer, while the bottom layer functions as an electromagnetic reflector. The upper graphene layer enhances the tunability of the converter, with its corresponding Fermi energy level denoted as *μ*_1_. The lower graphene layer selects polarization waves in specific directions, with its corresponding Fermi energy level denoted as *μ*_2_. By simultaneously adjusting the Fermi energy levels of the two graphene layers, the following three states are defined: *μ*_1_ = 0 eV and *μ*_2_ = 0.5 eV as State 1, *μ*_1_ = *μ*_2_ = 0.5 eV as State 2, and *μ*_1_ = *μ*_2_ = 1 eV as State 3.

To investigate the tunability of the proposed PCM, simulations were conducted to assess the polarization conversion performance with *μ*_1_ and *μ*_2_ set to 0 eV, 0.5 eV, and 1 eV under y-polarized incident waves. The polarization conversion ratio (*PCR*) is a crucial parameter for evaluating the polarization conversion performance of the metasurface, defined as follows [32]:

(5) $$PCR=\frac{r_{\mathrm{xy}}^{2}}{r_{xy}^{2}+r_{yy}^{2}}$$

In (5), *r*_xy_ is the co-polarized reflection coefficient, and *r*_yy_ is the cross-polarized reflection coefficient.

**Figure 2:**
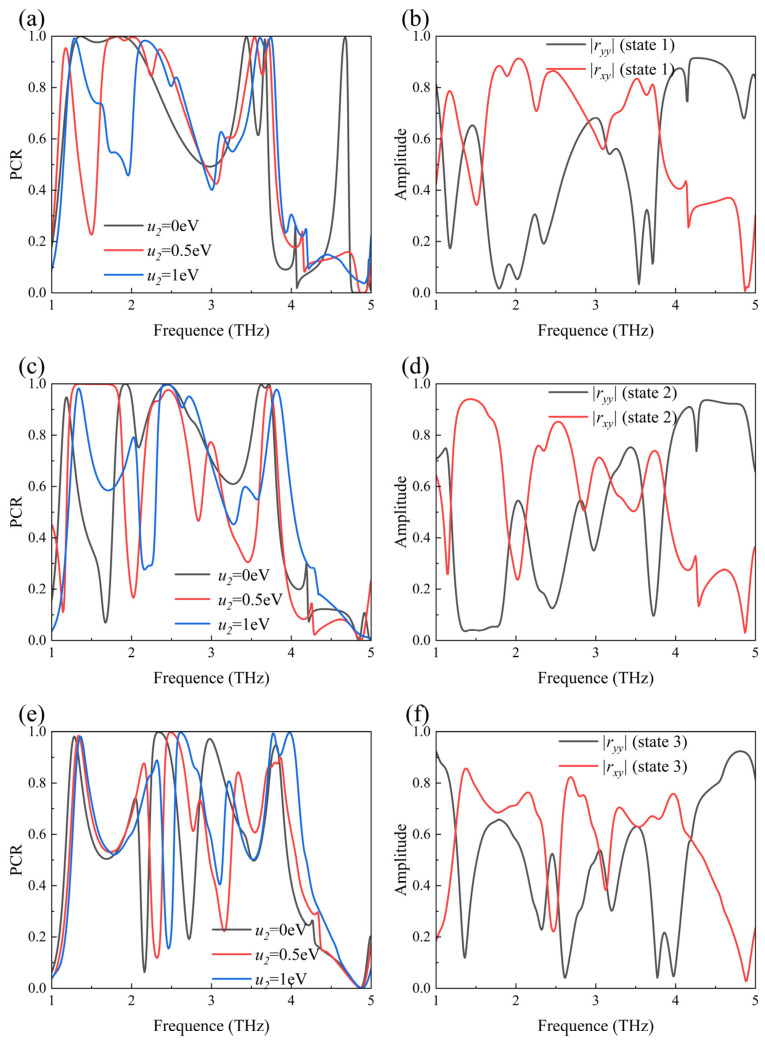
The polarization conversion ratio of the PCM unit at (**a**) *μ*_1_ = 0 eV, (**c**) *μ*_1_ = 0.5 eV, and (**e**) *μ*_1_ = 1 eV. The |*r*_yy_| and |*r*_xy_| amplitude of the PCM unit at (**b**) State 1 (*μ*_1_ = 0 eV, *μ*_2_ = 0.5 eV), (**d**) State 2 (*μ*_1_ = *μ*_2_ = 0.5 eV), and (**f**) State 3 (*μ*_1_ = *μ*_2_ = 1 eV).

Figure 2a,b present the data for State 1, where the PCR exceeds 0.9 in the ranges of 1.65–2.19 THz and 2.29–2.45 THz. Taking State 1 as an example, the process of polarization wave conversion is described in detail. As shown in Figure 2b, in the bands of 1.65–2.19 THz and 2.29–2.45 THz, *r*_yy_ is relatively small while *r*_xy_ is significantly larger, indicating that the y-polarized incident wave is converted into an x-polarized wave upon reflection by the PCM. The amplitude of the x-polarized wave is approximately 0.8 times that of the incident y-polarized wave, while the amplitude of the reflected y-polarized wave drops below 0.3. Therefore, when the PCR approaches 1 (*r*_xy_ = 1, *r*_yy_ = 0), it signifies that the y-linear polarized wave is converted into an x-linear polarized wave upon reflection. Conversely, when the PCR approaches 0 (*r*_xy_ = 0, *r*_yy_ = 1), it indicates that the y-polarized wave remains y-polarized after reflection. Figure 2c,d show the data for State 2, where the PCR exceeds 0.9 in the ranges of 1.23–1.85 THz and 2.24–2.60 THz. For State 3, Figure 2e,f demonstrate that the PCR exceeds 0.9 in the bands of 2.56–2.75 THz and 3.73–4.05 THz.

**Figure 3:**
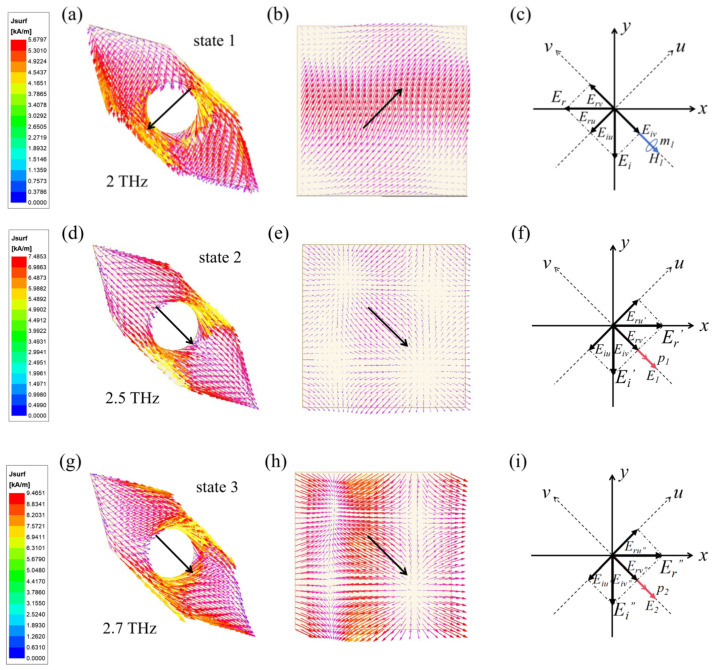
Surface current distribution on the top and bottom layers. The current directions in each layer are indicated by black arrows. (**a**,**b**) State 1 at 2 THz, (**d**,**e**) State 2 at 2.5 THz, and (**g**,**h**) State 3 at 2.7 THz. Equivalent electromagnetic moments of the incident and reflected waves at (**c**) 2 THz (State 1), (**f**) 2.5 THz (State 2), and (**i**) 2.7 THz (State 3).

**Figure 4:**
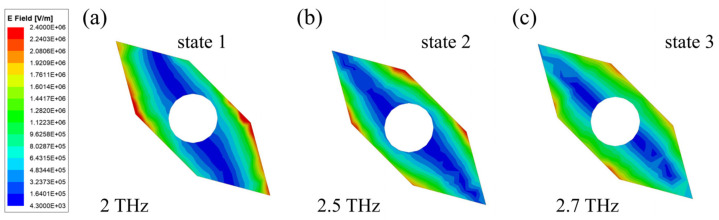
Electric field distribution at (**a**) 2 THz (State 1), (**b**) 2.5 THz (State 2), and (**c**) 2.7 THz (State 3).

To elucidate the physical mechanism of polarization conversion, Figure 3 shows the surface current distributions on the top and bottom metal layers at various resonant frequencies. The current directions in each layer are indicated by black arrows. To facilitate a better understanding of this mechanism, we randomly selected frequency points where the polarization conversion ratio (PCR) was close to one under different states, as shown in Figure 1 for our analysis. The chosen frequencies were 2 THz (State 1), 2.5 THz (State 2), and 2.7 THz (State 3). As illustrated in Figure 3a,b, the currents on the top and bottom layers flow in opposite directions, forming a closed loop that acts like a magnetic dipole and induces magnetic resonance. This magnetic dipole moment, labeled as *m*_1_, is depicted in Figure 3c. In contrast, Figure 3d,e show that when the currents on the top and bottom layers flow in the same direction, the interaction between the two layers generates an electric dipole, leading to electric resonance. The corresponding electric dipole moments, *p*_1_ and *p*_2_, are shown in Figure 3f,i. Both magnetic and electric dipole moments can achieve polarization conversion by adjusting the phase of the incident wave [33,34]. Using 2 THz (State 1) as an example, the linear polarization conversion process is explained in detail. The incident wave’s electric field (*E*_i_), aligned along the y-axis, is decomposed by the top layer into orthogonal components *E*_iu_ and *E*_iv_. As shown in Figure 3c, the surface currents on both layers are aligned along the u-axis, indicating that the *E*_iu_ component excites magnetic resonance without a phase change. The *E*_iv_ component, however, is reflected by the bottom metal layer, resulting in a 180° phase shift. Consequently, the reflected components *E*_ru_ and *E*_rv_ have an absolute phase difference of 180°, producing an x-polarized wave. The mechanisms for State 2 and State 3 are similar. To aid understanding, the electric field distributions are also provided, as shown in Figure 4. Through the above simulation analysis, the PCM can achieve a high PCR in different bands by adjusting the Fermi level of graphene. This prepares the groundwork for achieving tunable RCS reduction.

## 3. Application of PCM in Array Antennas

**Figure 5:**
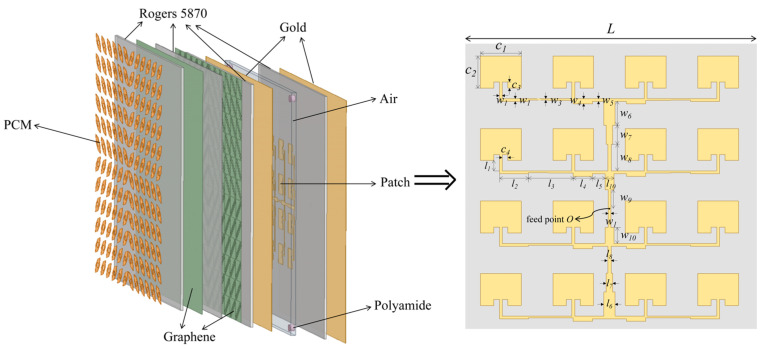
Schematic diagram of the overall structure.

The PCM is made up of 12 × 12 units, and the overall dimension is 720 × 720 μm^2^. Each quadrant contains 6 × 6 units arranged in a checkerboard pattern. The proposed PCM is placed above the array antenna to reduce its RCS, with a 14 μm air layer added between the array antenna and the PCM, which is supported by polyamide columns at the corners. The proposed antenna consists of a 4 × 4 microstrip antenna array. The specific structure is shown in Figure 5. The top metal patch and the ground plane (gold) each have a height of 1 μm, and the dielectric layer (Rogers 5870) has a height of 6 μm. This array antenna employs a coaxial feed method. To achieve better radiation performance and multi-frequency operation, the structure was optimized. The optimized geometric parameters are listed in Table 2. 

**Figure 6:**
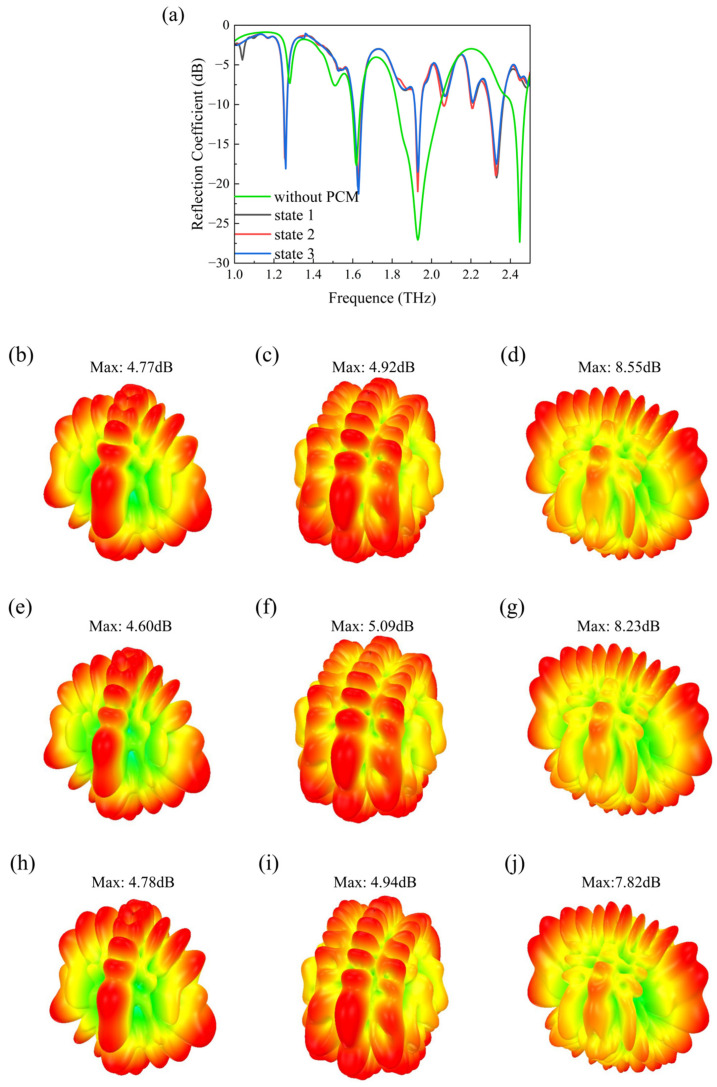
Comparison of the performance between the array antenna with and without PCM. (**a**) Reflection coefficient S_11_. The 3D radiation pattern at 1.63 THz for (**b**) State 1, (**e**) State 2, and (**h**) State 3. The 3D radiation pattern at 1.93 THz for (**c**) State 1, (**f**) State 2, and (**i**) State 3. The 3D radiation pattern at 2.30 THz for (**d**) State 1, (**g**) State 2, and (**j**) State 3.

To investigate the impact of the PCM on the radiation performance of the array antenna, the reflection coefficient S_11_ of the array antenna combined with the PCM was simulated. As shown in Figure 6a, the array antenna with the PCM exhibits three resonance frequencies at 1.62 THz, 1.93 THz, and 2.30 THz. The other graphs in Figure 6 show 3D radiation patterns at 1.62 THz, 1.93 THz, and 2.30 THz under different states. When the Fermi energy level changes, the gain of the antenna does not change significantly. Since metals can reflect electromagnetic waves, the presence of metallic materials near the antenna increases the loss resistance and reduces radiation efficiency. Therefore, compared to the array antenna alone, there is a slight shift in the resonance frequencies, but the antenna still maintains its radiation function.

**Figure 7:**
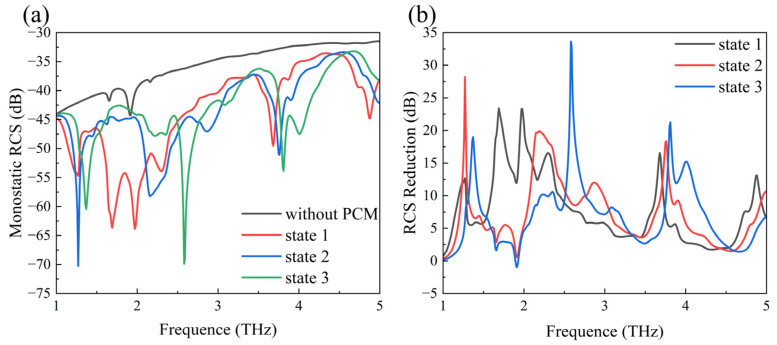
(**a**) Comparison of the monostatic RCS between the array antenna with and without PCM. (**b**) The RCS reduction.

To investigate the tunability of the proposed PCM for RCS reduction in the array antenna, Figure 7a presents the monostatic RCS under different Fermi energy levels, and Figure 7b more intuitively illustrates the RCS reduction effect. In State 1, the frequency bands with RCS reduction values greater than 10 dB are 1.60–2.43 THz and 3.63–3.72 THz. The maximum RCS reduction is 23.3 dB at 1.97 THz. In State 2, the RCS reduction values are greater than 10 dB in the bands of 2.07–2.53 THz, 2.78–2.98 THz, and 3.70–3.81 THz. The RCS reduction peaks are at 19.8 dB at 2.19 THz. In State 3, the frequency bands with RCS reduction values greater than 10 dB are 1.32–1.43 THz, 2.51–2.76 THz, and 3.76–4.13 THz. The maximum RCS reduction is 24.6 dB at 2.57 THz. Therefore, it can be concluded that the proposed PCM achieves significant tunable antenna RCS reduction by adjusting the Fermi energy level of graphene. It is worth noting that, according to current research on 2D materials, producing large-area uniform monolayer graphene remains a challenge [35,36,37,38,39].

The PCM proposed in this paper can be adjusted to three different states, offering significantly greater flexibility compared to previously reported results. Additionally, some engineering approaches using metasurfaces to reduce RCS in antenna can adversely affect the antenna’s radiative performance. In contrast, our proposed PCM maintains the antenna’s radiative performance, achieving a peak RCS reduction of over 19 dB in each state. Specific parameters for comparison are provided in Table 3.

## 4. Conclusions

In summary, to achieve tunable RCS reduction, this paper proposes and analyzes a graphene-based metamaterial polarization conversion unit. This unit comprises top-layer shuttle-shaped metal and two layers of graphene: the upper layer features a square pattern, and the lower layer features an inclined grating pattern. The proposed unit and its mirror image are arranged in a chessboard pattern to form the PCM. Experimental results demonstrate that placing this PCM above an array antenna can significantly reduce its RCS while maintaining basic radiation functions. Furthermore, by adjusting the Fermi energy level of the graphene, the PCM can achieve both an in-band and out-of-band tunable RCS reduction of more than 10 dB. This work potentially offers new insights into the development of switchable and tunable RCS reduction for antennas and related applications.

## Figures and Tables

**Table 1:** Specific geometric parameters of the polarization conversion unit.

Parameter	Length (μm)	Parameter	Length (μm)	Parameter	Length (μm)
*L*	60	*m*	28.28	*n*	24.49
*r*	8	*h*_1_	1	*h*_2_	8
*h*_3_	2	*h*	18	*d*	2
*d*_1_	3.92	*d*_2_	15		

**Table 2:** Optimized geometric parameters.

Parameter	Length (μm)	Parameter	Length (μm)	Parameter	Length (μm)
*C*_1_	58.12	*C*_2_	45.53	*C*_3_	8.5
*C*_4_	8	*l*_1_	15	*w*_1_	3.92
*l_2_*	41.35	*w*_2_	3.92	*l*_3_	63.87
*w*_3_	2.4	*l*_4_	27.27	*w*_4_	7.53
*l*_5_	16.4	*w*_5_	3.92	*l*_6_	15.92
*w*_6_	34.195	*l*_7_	9.26	*w*_7_	27.03
*l*_8_	4.62	*w*_8_	38.115	*l*_9_	3.92
*w*_9_	26.95	*l*_10_	12.86	*w*_10_	22.72

**Table 3:** Comparison of parameters from similar works in the references.

Ref.	Tunability	RCS Reduction Band	Maximum RCS Reduction	Impact on Antenna Performance
[26]	No	8–26 GHz	21.2 dB	Improved
[40]	No	0.4–0.575 THz	15 dB	Slightly decreased
[41]	Yes	ON-State 2–6 GHz OFF-State 1–8 GHz	27 dB /	Unchanged
This paper	Yes	State 1 1.60–2.43 THz 3.63–3.72 THz State 2 2.07–2.53 THz 2.78–2.98 THz 3.70–3.81 THz State 3 1.32–1.43 THz 2.51–2.76 THz 3.76–4.13 THz	23.3 dB 19.8 dB 24.6 dB	Unchanged

## Data Availability

The data that support the findings of this study are available from the corresponding author upon reasonable request.
